# How Doctors Generate Diagnostic Hypotheses: A Study of Radiological Diagnosis with Functional Magnetic Resonance Imaging

**DOI:** 10.1371/journal.pone.0028752

**Published:** 2011-12-14

**Authors:** Marcio Melo, Daniel J. Scarpin, Edson Amaro, Rodrigo B. D. Passos, João R. Sato, Karl J. Friston, Cathy J. Price

**Affiliations:** 1 Laboratory of Medical Informatics (LIM 01), Faculty of Medicine of the University of São Paulo, São Paulo, Brazil; 2 Department and Institute of Radiology (LIM 44), Faculty of Medicine of the University of São Paulo, São Paulo, Brazil; 3 Center for Mathematics, Computation and Cognition, Federal University of ABC, Santo André, Brazil; 4 Wellcome Trust Centre for Neuroimaging, University College London, London, United Kingdom; University of Groningen, Netherlands

## Abstract

**Background:**

In medical practice, diagnostic hypotheses are often made by physicians in the first moments of contact with patients; sometimes even before they report their symptoms. We propose that generation of diagnostic hypotheses in this context is the result of cognitive processes subserved by brain mechanisms that are similar to those involved in naming objects or concepts in everyday life.

**Methodology and Principal Findings:**

To test this proposal we developed an experimental paradigm with functional magnetic resonance imaging (fMRI) using radiological diagnosis as a model. Twenty-five radiologists diagnosed lesions in chest X-ray images and named non-medical targets (animals) embedded in chest X-ray images while being scanned in a fMRI session. Images were presented for 1.5 seconds; response times (RTs) and the ensuing cortical activations were assessed. The mean response time for diagnosing lesions was 1.33 (SD ±0.14) seconds and 1.23 (SD ±0.13) seconds for naming animals. 72% of the radiologists reported cogitating differential diagnoses during trials (3.5 seconds). The overall pattern of cortical activations was remarkably similar for both types of targets. However, within the neural systems shared by both stimuli, activation was significantly greater in left inferior frontal sulcus and posterior cingulate cortex for lesions relative to animals.

**Conclusions:**

Generation of diagnostic hypotheses and differential diagnoses made through the immediate visual recognition of clinical signs can be a fast and automatic process. The co-localization of significant brain activation for lesions and animals suggests that generating diagnostic hypotheses for lesions and naming animals are served by the same neuronal systems. Nevertheless, diagnosing lesions was cognitively more demanding and associated with more activation in higher order cortical areas. These results support the hypothesis that medical diagnoses based on prompt visual recognition of clinical signs and naming in everyday life are supported by similar brain systems.

## Introduction

There is substantial and converging evidence that a significant part of the understanding of the environment that we have in our everyday lives is carried out by brain mechanisms that are fast, automatic, and effortless [1], [2], [3]. Possibly as a consequence of these processes, diagnostic hypotheses in medical practice are often made by physicians in the first moments of contact with patients; sometimes even before the report of symptoms [4], [5], [6], [7], [8]. To exemplify, when a doctor encounters a patient with pronounced jaundice diagnostic hypotheses related to liver diseases immediately and automatically come to her/his awareness. This type of diagnosis has been ascribed to pattern recognition or non-analytical reasoning [9], [10].

**Figure 1:**
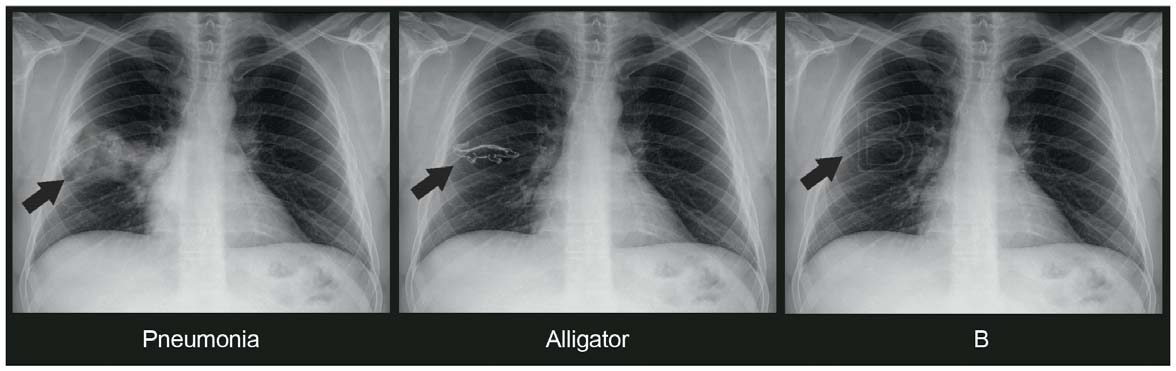
A trio of chest X-ray images with pairing of a lesion, an animal, and a letter*. *Arrows pointing to targets in the image; not present in the original images.

We propose that the generation of diagnostic hypotheses in such circumstances is the result of neurocognitive processes that are similar to those involved in naming objects or concepts in everyday life. Conversely, recognition of objects in everyday life can be conceptualized as a diagnostic process [11]. A critical test of this proposal would be to compare the brain systems involved in diagnosing lesions with those involved in naming. To explore this hypothesis, radiological diagnosis was used as a model in the visual domain. We developed an experimental paradigm in which radiologists diagnosed lesions in chest X-ray images and named non-medical targets (animals) embedded in chest X-ray images, during functional magnetic resonance imaging (fMRI). We expected to show that diagnosing lesions and naming animals, presented in the same context, would produce a similar pattern of brain activations. Naming letters was introduced as a control task (see Figure 1).

## Results

Mean response times (RTs), error and hesitation rates are shown in Table 1.

**Table 1:** Behavioural results.

Stimulus category	Response times* in seconds (SD)	Error rate# in percentage	Hesitation rate+ in percentage	Lexical semantic associations index⊕ (SD)
Lesions	1.33 (±0.14)	6.01	2.33	0.48 (±0.22)
Animals	1.23 (±0.13)	2.87	1.27	0.19 (±0.18)
Letters	1.14 (±0.13)	2.10	0.33	0.08 (±0.12)

*excluding outliers, repeated measures ANOVA F [Greenhouse-Geisser correction (GGc)] (1.54, 37.01) = 127.52 p<0.001; least significant difference (LSD) lesions>animals p<0.001, lesions>letters p<0.001, animals>letters p<0.001. Outliers percentage: lesions = 5.00%, animals = 1.27%, letters = 0..33%.

# repeated measures ANOVA F(GGc) (1.23, 29.59) = 12.73 p = 0.001; LSD lesions>animals p = 0.007, lesions>letters p<0.001, animals>letters p = 0.110.

+ repeated measures ANOVA F(2, 48) = 11.01 p<0.001; LSD lesions>animals p = 0.039, lesions>letters p<0.001, animals>letters p = 0.010.

⊕ mean number of words per stimuli type (see text and Appendix for further details); repeated measures ANOVA F(2, 48) = 49.57 p<0.001; LSD lesions>animals p<0.001, lesions>letters p<0.001, animals>letters p = 0.002. Numbers are from 24 subjects because one participant was incompletely debriefed.

When naming lesions, subjects reported becoming aware of a greater number of potential names in comparison to animals and letters (as evidenced by the lexical semantic association indices in Table 1). The associated words were usually different names, i.e. synonyms, for the same target, e.g. ‘enlarged heart’ while diagnosing cardiomegaly, or the two words name for the lesion, e.g. ‘mediastinal enlargement’. However, 18 (72%) participants reported that while diagnosing some lesions the name of alternative diagnoses came to mind, e.g. ‘bulla’ while diagnosing a cavitation; 15.8% of the lexical semantic associations for lesions were differential diagnoses. Twenty-two (88%) participants reported becoming aware of the name of other animals as alternatives to some of the animals they were naming; e.g. ‘dromedary’ while naming a camel; 64.0% of the lexical semantic associations in this category were the names of other animals.

In 8.00% of the correct responses for lesions, subjects responded with a one-word name other than the name learned during training (e.g. ‘condensation’ in response to pneumonia). Also in 5.13% of lesion trials, the correct responses had more than one word (e.g. ‘aortic elongation’ or ‘pleural effusion’).

**Figure 2:**
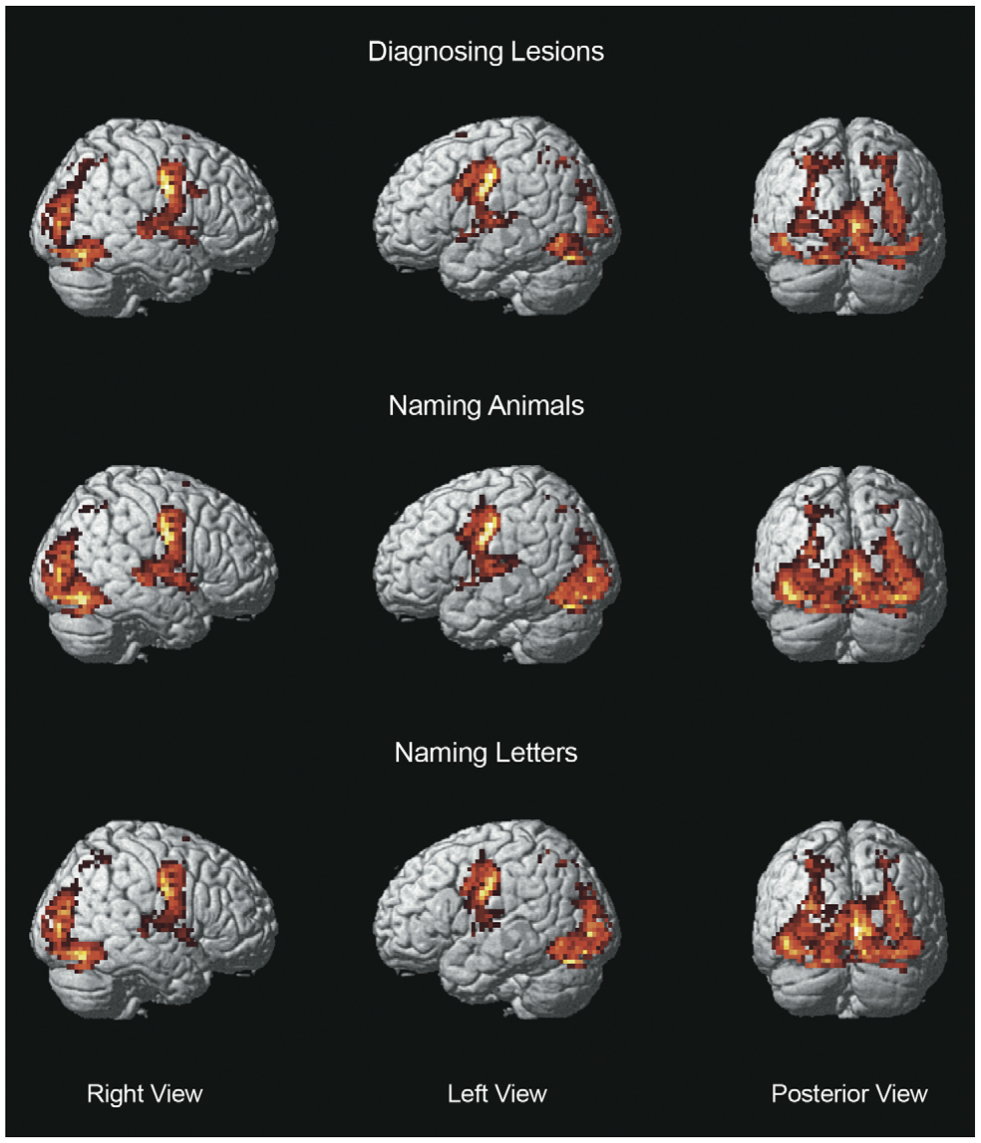
Cortical activations while diagnosing lesions and naming animals and letters versus control baseline *. *family wise error rate corrected p<0.05. Statistical parametric maps rendered on an International Consortium of Brain Mapping individual brain.

**Figure 3:**
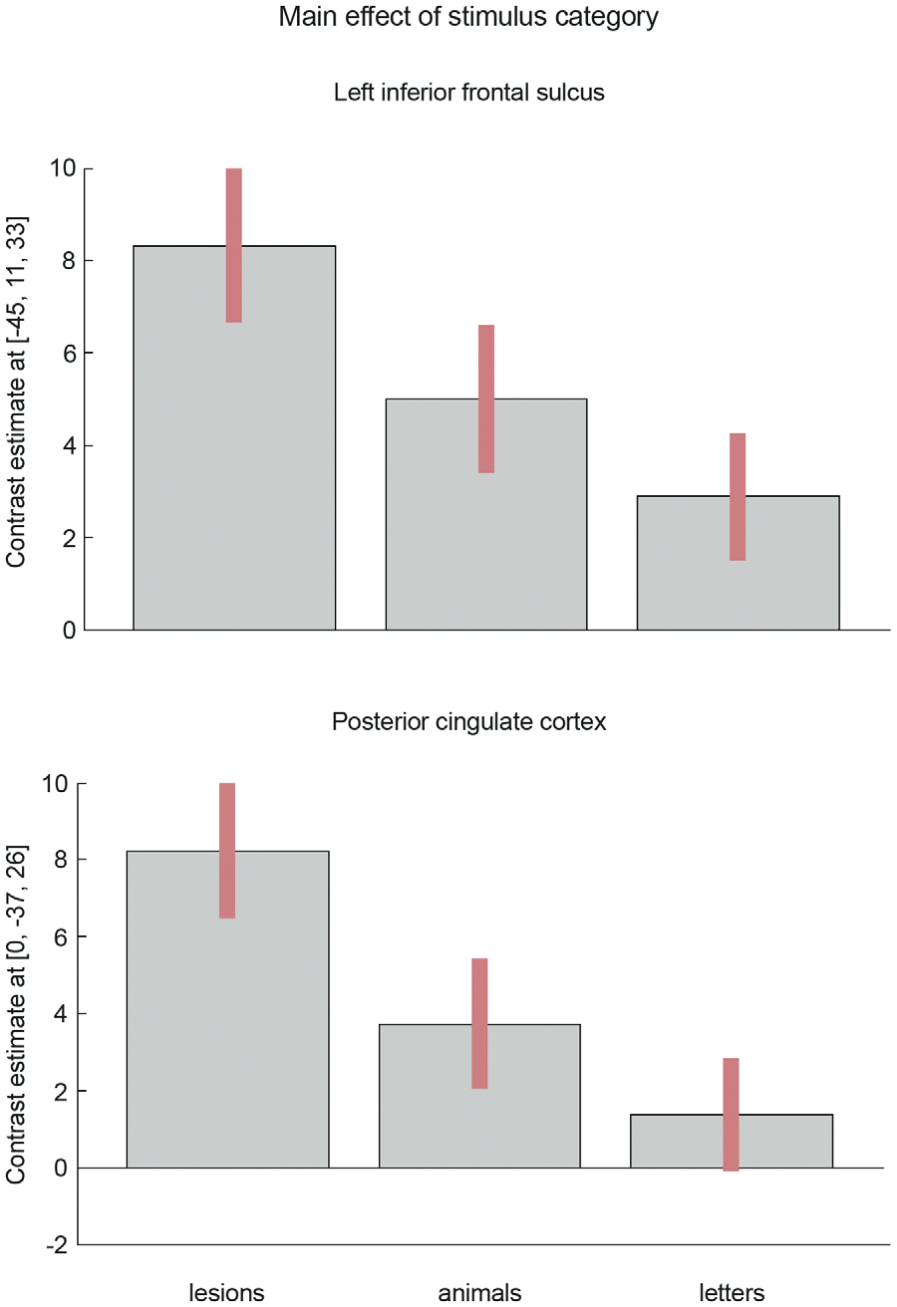
Contrast estimates* in cortical areas more active for diagnosing lesions^#^. * 90% confidence interval. # contrast [lesions>(animals and letters)] inclusively masked with lesions>baseline, lesions>animals, and lesions>letters at p = 0.001.

The patterns of cortical activations observed when naming each category of stimulus (relative to a control baseline of null events), were strikingly similar in their anatomical deployment (Figure 2). When lexical semantic associations were not controlled, activation was higher for naming lesions than naming animals and letters in the left inferior frontal sulcus and posterior cingulate cortex (Table 2 and Figure 3). Activation in the same areas was also higher for naming animals than naming letters (p<0.001 uncorrected). This decreasing order of activation: lesions>animals>letters (Figure 3) is parallel to a similar order of diminishing lexical semantic association indices (Table 1). Indeed, when lexical semantic associations were co-varied out in the second statistical analysis, there were no areas where activation was significantly higher for lesions than animals and letters. This contrasts to the activation in more posterior regions, posterior fusiform gyrus and posterolateral occipital cortex, that was higher for animals than lesions (Table 2 and Figure 2).

**Table 2:** Contrasts between diagnosing lesions and naming animals and letters *.

Anatomical region	Local maxima activations (MNI coordinates x, y, z)	z+ (peak level)	Cluster size# (voxels)
lesions>animals and letters**			
left inferior frontal sulcus	−45 11 32	5.92	33
posterior cingulate gyrus	0 −37 26	6.93	21
	0 −25 29	5.32	
animals>lesions¶			
right posterior fusiform gyrus and posterolateral occipital cortex	45 −79 −4	>7.82	139
	39 −52 −20	7.82	
	39 −85 −7	7.77	
left posterior fusiform gyrus and posterolateral occipital cortex	−45 −79 −1	>7.61	137
	−36 −55 −17	>7.61	
	−39 −79 −10	7.61	

*familywise error rate corrected p<0.05.

+ significant at p<0.001.

# extent threshold: 10 voxels.

**inclusively masked by lesion>baseline, lesion>animal, and lesion>letter at p = 0.001.

¶ inclusively masked by lesion>baseline and animal>baseline at p = 0.001.

In summary, naming lesions, animals and letters activated the same set of distributed brain regions but to significantly different degrees. In high-order cortical regions (prefrontal and cingulate cortices), activation was proportional to the number of lexical semantic associations (lesions>animals>letters), while in visual cortices activation was higher for animal naming.

## Discussion

This investigation was conducted to test the proposition that generation of diagnostic hypotheses evoked by the immediate visual recognition of clinical signs engages neural systems that are recruited when naming objects in everyday life. The results support this hypothesis by showing very significant and similar activations in a circumscribed set of distributed cortical regions when naming radiological lesions and animals in the same context. However, diagnosing lesions was cognitively more demanding and associated with more activation in higher order cortical areas.

Higher mean RTs, error, and hesitation rates suggest that, on average, diagnosing lesions was more difficult than naming animals in our experimental setting. This could be related to the visual characteristics of lesions and/or the fact that low-frequency (in everyday language) lesion names are more difficult to recall, compared to high-frequency animal and letter names [12]. Lexical semantic associations, i.e. being aware of words/concepts other than that vocalized, were more frequent while diagnosing lesions (Table 1), as compared to the other two categories, also indicating greater cognitive demand related to the selection of appropriate names in this particular task.

A relevant aspect of our results was that generation of diagnostic hypotheses can be very fast; the mean RT to diagnose lesions was 1.33 seconds. It is possible that the training before the fMRI experiment contributed to this performance. But very rapid identification of lesions (<1 second) has already been reported in radiology studies [13], [14], [15].

An important finding was that radiologists were able to cogitate differential diagnoses during the 3.5 seconds of a trial. A similar awareness of alternative names of other animals was reported while naming animals. In a few cases even letters evoked words to subjects (Table 1). Participants were not instructed to make differential diagnoses or to think about alternative names for animals during the task. These results are compatible with a fast and automatic semantic association process, in which the recall of a diagnosis or a name of an animal occurs with the concomitant activation of semantically related concepts [16], [17], [18]. Clearly, a formal and definitive diagnosis can not be made in seconds but its core cognitive process, the generation of diagnostic hypotheses which were the names of lesions in our study, is crucial for a final correct diagnosis [4], [5], [6], [7], [8].

Diagnosing lesions in radiological images can be conceptualized as a process that is similar to localizing and naming objects in a scene [19]. There are several fMRI studies of location and recognition [20] or naming objects [21] but we are unaware of studies in which all of these tasks are combined. We found very significant brain activations associated with the localization, recognition and naming of targets in radiological images, be they lesions, animals, or letters (Figure 2). Activations were greater in the left inferior frontal sulcus and posterior cingulate cortex for naming lesions than animals and also greater for naming animals than letters (Table 2 and Figure 3). When lexical semantic associations, more frequent for lesions (Table1), were taken into account in an analysis of covariance the difference of activations between those regions was no longer significant (at a corrected level). All the regions activated in the present study have also been reported in object naming in other studies [21], therefore there was no indication that the participating radiogists were naming animals differently from laypeople.

In agreement with our findings, activation in the left inferior frontal sulcus was reported in two fMRI studies of semantic verbal fluency - generating and vocalizing associated words in response to a word - in which there was greater lexical semantic demand in contrast to the comparison task, reading aloud [22], [23]. This is consistent with increased cognitive control when it is necessary to make a choice between synonymous or competing concepts, e.g. synonym words or differential diagnoses in our study, respectively [24].

There were regions more activated by naming animals relative to diagnosing lesions. These regions are usually associated with visual processing and recognition of stimuli; namely, posterior fusiform cortex and posterolateral occipital cortex (Table 2 and Figure 2) [25]. We believe that differences in the visual characteristics of the stimulus categories we used are responsible for the observed differences in activations in these cortical areas.

The cognitive mechanisms underlying medical diagnosis have been studied with different conceptual strategies [10], [26], [27]. One important approach relevant to the present study considers it a classification process similar to categorization in everyday life and several authors investigated diagnostic processes with different categorization models [28], [29], [30], [31], [32], [33], [34]. These studies explored several aspects of the cognitive psychology of diagnostic reasoning but did not include a comparison of medical diagnosis tasks with, e.g., categorization of objects.

Interestingly, from a historical point of view, similarities between the classification of diseases and living creatures have been suggested in the past. In the XVII^th^ century Thomas Sydenham in his influential definition of diseases proposed to conceptualize them as specific (ontological) entities similar to plant species [35]. Influenced by Sydenham ideas, Boissier des Sauvages created a classification of diseases, nosology, based on taxonomic principles used in botany and his approach was followed by other phyisicians in the XVIII^th^ century, including Carl Linnaeus [36].

Instead of using categorization, a concept with different meanings and diverse and conflicting models [37], [38], [39], we chose naming as a conceptually more prudent and descriptive approach. Picture naming has been a model extensively used in cognitive psychology [40], [41], [42], [43], and functional neuroimaging [21] to investigate how objects are recognized and named.

Some cognitive processes underlying medical skills have been studied with fMRI: one study investigated the neural substrate of visuo-spatial skills in surgery residents [44] and the other compared brain activations in radiologists versus lay participants while viewing X-ray images [45]. However, the neural basis of the medical diagnostic processes per se has not been investigated before.

Our experimental design was not planned to assess the generation of differential diagnostic hypotheses. Taking into account the limitations of the cued retrospective recall employed in the study, the conclusions resulting from the lexical semantic associations data need to be replicated using other methodological approaches.

In contrast to naturalistic and observational studies, planned experiments are by definition artificial and reductionistic due to the need to limit and control independent variables. Radiologists in their usual practice do not usually vocalize their diagnostic hypotheses as they come to their awareness. Conversely, we do not habitually vocalize the names of objects as we recognize them in our everyday life.

Radiologists customarily verbalize their diagnosis using more than one word, e.g. pleural effusion in right hemithorax, in contrast to one-word names for animals. This difference in length of responses could introduce an important confound variable [46], [47]. To circumvent it, we trained the participants to preferentially use one-word names to diagnose lesions (see Methods and Appendix S1 for details). Probably as a consequence of this experimental stratagem the complete names of the lesions were reported to came to the awareness of participants and were considered competing lexical-semantic associations (see Results).

Under the blanket rubric ‘medical diagnosis’ there are different cognitive tasks and processes. Considering the case in point of radiological diagnosis: The immediate recognition and diagnosis of an obvious lesion probably recruits different neurocognitive processes as compared to the diagnosis of a subtle and ambiguous alteration with complex differential diagnoses requiring a detailed examination of the radiological image. To create our experimental design we had to limit the scope of the investigation and the conclusions of our study are restricted to the diagnosis of lesions that are immediately identified and diagnosed. It will be important to replicate these results with other approaches, e.g. electrophysiological methods such as electroencephalography or magnetoencephalography. The conceptual hypothesis also needs to be tested in other medical specialties in which diagnosis is strongly based on visual clinical data, e.g. dermatology.

This study is an attempt to investigate the brain mechanisms subserving medical diagnosis. We have demonstrated that differential diagnoses can be automatically elicited in a time frame of seconds in response to clinical signs. Our results support the hypothesis that a process similar to naming things in everyday life occurs when a physician promptly recognizes a characteristic and previously known lesion. In our experimental model, the diagnostic task was cognitively more taxing; more activation in higher order cortical areas was plausibly associated with demands related to the selection of appropriate names as compared to the control task.

The importance of non-analytical reasoning in medical diagnosis has been increasingly stressed [9], [10], [27], [48]. Our study is a contribution to the understanding of its mechanisms. There are recent reviews proposing the application of the knowledge acquired in neuroscience to improve medical education methods [49], [50], [51]. An implication of our results is that information obtained from cognitive neuroscience studies on the recognition and naming of objects can be brought to bear on the improvement of diagnostic expertise in the visual domain. In addition, the conceptual hypothesis and the methodological approach described in the present investigation may open new ways to develop studies in medical diagnosis.

## Materials and Methods

### Participants

Twenty-six radiologists participated in the investigation. One subject was excluded because the responses were not recorded due to technical problems. Inclusion criteria were completion of radiology residency, right-handedness (as assessed by a modified version of the Edinburgh Handedness Inventory [52]), and Portuguese as the native language; exclusion criteria were neurological and psychiatric disorders. Sixteen participants were male. The mean age of subjects was 35.9 years (range: 27–55), with a mean of 11.6 years of radiological practice (range: 4–30).

### Ethics statement

The protocol was approved by the research ethics committee of the Clinics Hospital, Faculty of Medicine of the University of São Paulo, Brazil. All participants gave written informed consent. They did not receive monetary compensation for their participation.

### Radiological images

Our experimental design required the radiological images to have just one circumscribed visual target that could be named. Since many thoracic radiological lesions co-occur, e.g. cardiomegaly is commonly associated with radiological signs of pulmonary venous congestion, we embedded lesions in normal X-ray images using image editing software.

Twenty different types of thoracic radiological lesions, with six different exemplars of each, were created. We used clearly identifiable and easily diagnosable lesions to minimize expertise confounds at the between-subject level and to ensure ceiling performance (to preclude performance confounds). The face validity [53] of radiological images with lesions was assessed by two senior thoracic radiologists. To create non-medical targets line drawings of animals were superimposed on the radiological images. These targets were selected from the database of the International Picture Naming Project [54]. Each type of animal, with six different exemplars, was paired with one type of lesion. Finally, 20 different consonant letters, each with six exemplars from different fonts, were paired with each type of lesion. The resulting radiological images comprised six sets of 60 different stimuli: 20 with lesions, 20 with animals, and 20 with letters.

Longer words or naming targets with more than one word might be associated with longer response times and different patterns of brain activations in regions involved in language processing [46], [47]. To control for this confounder, we created a list of one-word names to diagnose lesions, e.g. ‘effusion’ for pleural effusion, and asked subjects to use these terms. In additon the duration of vocalization of the radiological lesion names was paired to that of the animal names. Searching for lesions, animals, and letters with the accompanying eye movements was an important variable. We controlled for it matching the locations of the three types of alterations in the chest X-ray images. The methodology used to create the radiological images is detailed in Appendix S1.

There are many subtleties in a veridical radiological lesion that can not be reproduced with image editing software. For this reason the lesions we created can be considered caricatures of true lesions in the same way that line drawings are an iconic representation of animals.

The key differences between the three categories of stimuli comprised: 1- the visual attributes of lesions where most lesions had simpler and more heterogeneous forms compared to animals and letters which had more defined and homogeneous contours (see figure 1); 2- despite the absence of quantitative data on word frequency in medical domain, probably medical terms have in general a lower frequency in daily language and an older age of acquisition in comparison to animals and letters.

### The experiment

The creation of images with just one target and the short viewing time of the stimulus images were critical points of our experimental strategy; they were intended to block a more careful scanning of the radiological images, which normally occurs in radiological practice. The neurocognitive processes involved in detailed scanning of the image and images with different numbers of targets to name would be important experimental confounders.

Radiological stimuli were projected through a magnetic shielded glass window to a screen inside the scanner room using a Dell 2400MP digital slide projector. The stimuli subtended 12.5° horizontal and 9.4° vertical visual angles. Each image was presented for 1.5 seconds. Radiologists were oriented just to name the target (lesion, animal or letter) as soon as it was recognized. The task implicitly involved localizing the target, recognizing it, retrieving its name and articulating the response [21], [55], [56]. Every image presentation was followed by a black screen with a white (central) fixation cross for 2.0 seconds (i.e., 3.5 seconds per trial). This design was optimized during pilot testing to minimize trial duration, while preserving near-ceiling performance. Participants were trained immediately before the scanning session with three different sets of images.

We used an event-related design: In each session, there were 60 trials (20 lesions, 20 animals, and 20 letters) and 20 null events (with just a fixation cross). There were three sessions per participant with three different sets of images to preclude perceptual learning, repetition suppression and other adaptation effects confounding the naming related responses. Three different sequences of stimuli presentation were optimized in terms of the efficiency to disclose fMRI responses using a genetic algorithm [57]. Each of the three sets of images was presented with one of the three optimal sequences. The order of the sequences was counterbalanced over subjects. The image sets and the order of their presentation for training and scanning were also counterbalanced between participants.

There were two control conditions: 1- naming letters, a high-level baseline, with all cognitive components of the task in diagnosing lesions and naming animals except word retrieval; 2- null events intermixed with stimuli, with a fixation cross during 3.5 seconds, during which participants had no task to execute.

Response time was defined as the elapsed time between the stimulus onset (image presentation) to the beginning of vocalization of the response. Error was defined as error proper or no response. Hesitations were considered as: 1- beginning to vocalize a word and changing to another or 2- stammering on the beginning of the vocalization.

Words and concepts may have different numbers of other words and concepts semantically associated to them [18]. There are indications that the number of potential associates to a word may influence the pattern of brain activations in tasks involving word production [58]. To control for this variable subjects were debriefed immediately after the experiment to assess lexical semantic associations - words other than that vocalized that came to their awareness while naming the different types of stimuli - in each type of stimulus following a standardized protocol (see Appendix S1). Participants were not informed beforehand of the debriefing protocol.

Retrospective recall has limitations [59] but we could not monitor those associations during fMRI data acquisition. To instruct subjects to report them after each stimulus could induce participants to actively search for associated words and concepts and create an important confounder in the experimental design. However, classical memory studies investigating cued recall found very high recall rates in tasks more demanding than in our investigation in particular when there was semantic processing while encoding the stimuli [60], [61], [62]. Also, retrospective recall methods have been used as reliable assessments in the context of fMRI experiments [63], [64].

### Data acquisition

MR images were acquired in a 3 Tesla Philips Achieva system with an 8-channel head coil. Blood oxygenation level-dependent (BOLD) sensitive T2*-weighted images were obtained using an SENSE gradient-echo echo-planar imaging pulse sequence with the following parameters: repetition time: 2500 ms, echo time: 30 ms, flip angle: 90°, field of view: 240 mm^2^, in-plane voxel resolution: 3 mm^2^. Fifty 3 mm axial slices were acquired, with a slice gap of 0.3 mm and a +30° image plan tilt to reduce artifacts in inferior temporal lobe [65]. Functional sessions were preceded by 10.0 s of dummy scans to ensure steady-state magnetization. A T1-weighted structural image (voxel size: 1 mm^3^) was acquired after the functional sessions for coregistration with the fMRI data.

Stimulus presentation and response recording were performed with E-Prime 2.0 software (Psychology Software Tools). A plastic mouthpiece was anatomically adjusted to the mouth of the participants enabling to isolate the sound of the vocalization of responses from the scanner's noise. The voice sound was conducted through a pneumatic system to a high-sensitivity microphone outside the scanner room, pre-amplified, and recorded. Barch et al. described a similar approach to record overt verbal responses [66]. Response times were measured following a standardized protocol using Praat 5.1, a software for phonetic analysis [67], after filtration of the background noise.

### Statistical and data analyses

Data processing and statistical analyses were conducted using SPM8 software (Wellcome Trust Centre for Neuroimaging) [68]. Functional volumes were realigned, un-warped, coregistered to the structural image, normalized to the MNI space, and smoothed with an isotropic Gaussian kernel with 6 mm FWHM.

After preprocessing, a first-level analysis was conducted at the individual level to estimate category-specific activations at each voxel. Time-series from each voxel were high-pass filtered with a cut-off period of 1/128 Hz to remove signal drift and low-frequency noise. A gray-matter image resulting from the segmentation of the structural image was used as a mask in the analysis of functional activations. All category-specific (lesion, animal or letter) trials were modeled as a stick-function and convolved with a canonical hemodynamic response function. Trials with errors, hesitations, and outlying RT's (>2 standard deviations of the mean RT for the respective target type) were modeled as events of no interest. RT's were also included as nuisance variables to remove response time effects within condition.

Category-specific activations were estimated in the usual way using the appropriate t-contrast. The resulting subject-specific contrast images were then entered into a second-level analysis of covariance (ANCOVA). This (random effects) between-subject analysis was conducted without and with lexical semantic associations (the mean number of lexical semantic associations for each category made by every participant) as a nuisance variable. The differences in category-specific activation were then assessed using statistical parametric maps (SPMs) with a criterion of *p*<0.05 (corrected for multiple comparisons using random field theory).

To limit head movements during the experiment participants were trained before the scanning session. The un-warping of the functional images during preprocessing with SPM8 was an additional measure to compensate for movements during the vocalization of responses [69]. Head movements during fMRI sessions were generally small, with intra-session translation and rotation movements of less than 1.5 mm and 1.5° respectively.

#### Supporting information

The methodology for the creation of radiological images with embedded targets, training and debriefing protocols are detailed in Appendix S1.

## Supporting Information

Appendix S1 (DOC)
