# Supplementary material for: How Doctors Generate Diagnostic Hypotheses: A Study of Radiological Diagnosis with Functional Magnetic Resonance Imaging
Source: PLoS One. 2011 Dec 14;6(12):e28752. doi: 10.1371/journal.pone.0028752 (PMC3237491; doi:10.1371/journal.pone.0028752)
Supplement: Appendix S1 — (DOC) [file pone.0028752.s001.doc]

**Appendix**

**1- Creation of images with radiological lesions**

**2- Creation of images with embedded animals**

**3- Creation of images with embedded letters**

**4- Pairing of the different stimuli**

**5- Training protocol**

**6- Debriefing protocol and calculation of the lexical semantic index**

**1- Creation of images with radiological lesions**

Only normal plain chest X-ray images of adult males in postero-anterior (PA) projection were used to have a more homogeneous set of stimuli from the visual point of view. A selection of 120 X-ray normal images from different subjects were employed to create the stimuli. Images were initially processed to reduce disparity in the contrast levels in the original digital images; a gray scale level of 230 (RGB color scale) was established to control for brightness.

Six different exemplars of each lesion were created using Photoshop CS4 Version 11 (Adobe Systems); each one in a different X-ray image. Six sets of 20 different types of lesions, comprising 120 radiological images, were assembled.

The magnetic shield mesh in the window through which the images were projected into the scanner room caused degradation of sharpness and contrast of the images; to compensate for that we used the 'unsharp mask' tool of Photoshop. The final step was to adjust brightness and contrast of radiological alterations in each image using as parameters blackness in the centre of the lungs and distinctiveness of the pulmonary hila, taking care to avoiding a pop-out effect. The adequacy of the images was assessed in the validation process (see below).

To assess their face validity, the images were presented to two experts in thoracic radiology who did not participate in the creation of the images. We elaborated three Likert-type scales to standardize their assessment of: 1- the difficulty to locate the lesion ; 2- the difficulty of the diagnosis; 3- the compatibility of the alteration with the prototype that the judge had of the abnormality. We defined as acceptance criteria that both judges agreed with that: 1- the lesion was 'very easily' or 'easily' located; 2- the lesion was 'very easily' or 'easily' diagnosable; 3- the lesion was in 'very strong' or 'strong' agreement with the mental prototype(s) or exemplar(s) that the judges had of it.

We created images with 29 different diagnoses: 1-pneumothorax, 2-pleural effusion, 3- pulmonary nodule, 3-pulmonary granuloma, 4-pneumonia, 5- rib fracture, 6- clavicule fracture, 7- pleural calcification; 8- aortic calcification; 9- giant pulmonary bulla; 10- cardiomegaly; 11- tracheal stenosis; 12- hiatus hernia; 13- tuberculosis; 14- pneumomediastinum; 15- aortic aneurism; 16- aortic calcification; 17- aortic elongation; 18- laminar atelectasis; 19- elevation of the diaphragmatic cupule; 20- azigos lobe; 21- megaesophagus; 22- cardiac pacemaker; 23- tracheal cannula; 24- mediastinal widening; 25- thoracic drain; 26- pulmonary cavitation; 27- tracheal deviation; 28- mediastinal tumor; 29- mitral valve prosthesis.

During the validation process, nine diagnoses were excluded: rib fracture, tracheal stenosis, tracheal deviation, cardiac pacemaker, megaesophagus, pneumomediastinum, pulmonary granuloma, aortic aneurism, and mediastinal tumor.

Strictly speaking tracheal cannula, mitral valve prosthesis, and thoracic drain are medical devices not lesions. Also, azigos lobe is a normal anatomical variant not a lesion. But for the sake of simplicity we subsumed them under the ‘lesions’ rubric.

When compatible with the character of the lesion, the alterations in each set of six images of the same alteration were distributed in the right and left hemithoraxes and in the different zones of the lungs in a counterbalanced way:

- pneumothorax: three in the right and three in the left lateral wall extending from the superior to the apical zone;

- pulmonary nodule: three in the left and three in the right lung; one in the upper, one in the middle, and one in the inferior zones;

- pneumonia: three in the left and three in the right lung distributed in the middle and inferior zones;

- clavicle fracture: three in the right and three in the left;

- pleural calcification: three in the right and three in the left lateral wall;

- giant pulmonary bulla: three in the left and three in the right lung; one in the upper, one in the middle, and one in the inferior zone;

- tuberculosis: three in the right and three in the left lung extending from the superior to the apical zones;

- laminar atelectasis: three in the left and three in the right lung distributed in the middle and inferior zones;

- elevation of the diaphragmatic cupule: three in the right and three in the left;

- thoracic drain: three in the right and three in the left;

- pulmonary cavitation: three in the left and three in the right lung; one in the upper, one in the middle, and one in the inferior zones;

The other lesions had central locations or in the case of azigos lobe had an obligatory upper right hemithorax localization.

Visual angles of radiological alterations ranged from an average of 0.2° horizontal (h) x 0.3°vertical (v) for pulmonary nodules to 2.9°(h) x 1.6°(v) for cardiomegalies.

**2- Creation of images with embedded animals**

We selected the animals from 66 line drawings of different animals of the International Picture Naming Project (IPNP) database (http://crl.ucsd.edu/~aszekely/ipnp). The initial selection criteria were: 1- viability of its insertion in the X-ray from the image processing point of view; 2- pairing the names of animals with the names of radiological alterations.

Sets of six different exemplars of each animal were created; one drawn from the IPNP database and five more created by us. In the end there were 6 sets of 20 different animals comprising 120 radiological images.

The drawing of animals complied to the following criteria:

- they should be perceived as different exemplars of the same animal and should evoke the same name;

- be preferentially in side view, three turned to left and three turned to right; octopuses and butterflies were two exceptions to this criterion;

- the drawings should avoid postures that could suggest movement including open mouths.

Each animal was positioned in the same location of the paired radiological alteration. The same normal thoracic image used to create a radiological lesion was used to embed the animal paired to that lesion.

The final criterion to create the pairing was the duration of the vocalization of both the radiological lesion and the animal name. We conducted a reading task to measure the duration of the vocalizations. The names of animals and radiological lesions were presented to ten radiologists in a computer screen using E-Prime 2.0 software (Psychology Software Tools) and their responses were recorded. Each name was presented three times in randomized order (see results below).

Radiological images with twenty six animals were pilot tested with radiology residents: rhinoceros, hippopotamus, porcupine, crab, elephant, butterfly, ostrich, turtle, scorpion, dolphin, bird, alligator, ant, monkey, mouse, dog, owl, kangaroo, horse, shark, camel, penguin, octopus, tiger, giraffe, whale. After pre-testing six animals were excluded: hippopotamus, crab, scorpion, giraffe, mouse, and whale.

As a consequence of different patterns of contours it was not possible to compatibilize the dimensions of animals and radiological lesions. To uniformise the size of animals in the radiological image we established that their height and width would define a rectangular area ranging from 8,000 to 10,000 pixels in a 1024x768 screen. This way visual angles ranged from a minimum of 0.3° to a maximum 0.8° in the horizontal axis and from a minimum of 0.3° to a maximum of 0.7° in the vertical axis.

**3- Creation of images with embedded letters**

Twenty consonant letters in upper or lower case were selected: B, C, D, F, G, h, J, K, L, m, n, p, q, r, S, T, v, W, x, Z. Vowels were excluded because they have a different neural processing when compared to consonants. Each letter was paired with one type of radiologial alteration and the same images used to create radiological lesions were used to embed the corresponding letters. The pairing was carried out taking into account the form of the letter (in upper or lower case) to enable it to be located in the same region and cover approximately the same area of the radiological lesion. Sets of six exemplars of the same letter in different fonts were created.

Visual angles of letters ranged from an average of 0.2°(h) x 0.3°(v) for p's to 1.6°(h) x 1.1°(v) for W's.

**4- Pairing of the different stimuli**

The final pairing of lesions, animals, and letters is presented in the table below:

| **Thoracic lesion** | **One word name** (in Portuguese) | **Duration of vocalization** (ms) (SD) | **Animal name** | **Duration of vocalization** (ms) (SD) | **Letters** |
| --- | --- | --- | --- | --- | --- |
| laminar atelectasis | atelectasia | 1,091 (±214) | rinoceronte  (rhinoceros) | 964 (±116) | v |
| aortic elongation | alongamento | 994 (±179) | avestruz  (ostrich) | 862 (±142) | C |
| tuberculosis | tuberculose | 978 (±159) | porco-espinho  (porcupine) | 859 (±109) | S |
| cardiomegaly | cardiomegalia | 976 (±112) | elefante  (elephant) | 852 (±95) | W |
| pneumothorax | pneumotórax | 958 (±148) | borboleta  (butterfly) | 843 (±86) | F |
| pleural calcification (pachypleuritis) | paquipleuris | 988 (±131) | tartaruga  (turtle) | 777 (±97) | J |
| mediastinal widening | alargamento | 890 (±163) | golfinho  (dolphin) | 770 (±19) | Z |
| diaphragmatic cupule elevation | elevação | 818 (±95) | passarinho  (bird) | 748 (±112) | m |
| pneumonia | pneumonia | 816 (±116) | jacaré  (alligator) | 711 (±102) | B |
| pulmonary cavitation | cavidade | 773 (±105) | formiga  (ant) | 687 (±108) | x |
| aortic calcification | ateroma | 735 (±108) | macaco  (monkey) | 667 (±91) | h |
| azigos lobe | azigos | 706 (±160) | cachorro  (dog) | 647 (±92) | r |
| mitral valve prosthesis | prótese | 674 (±109) | coruja  (owl) | 651 (±85) | D |
| clavicle fracture | fratura | 653 (±109) | canguru  (kangaroo) | 642 (±95) | n |
| pleural effusion | derrame | 623 (±82) | cavalo  (horse) | 638 (±95) | L |
| pulmonary nodule | nódulo | 588 (±97) | tubarão  (shark) | 594 (±81) | p |
| tracheal cannula | cânula | 583 (±111) | camelo  (camel) | 592 (±96) | T |
| giant pulmonary bulla | bolha | 543 (±82) | pinguim  (penguin) | 573 (±98) | G |
| hiatus hernia | hernia | 540 (±102) | polvo  (octopus) | 547 (±103) | K |
| thoracic drain | dreno | 485 (±64) | tigre  (tiger) | 547 (±85) | q |
|  | **mean** | **766 (±122**) | **mean** | **709 (±100)** |  |

**5- Training protocol**

The training of participants before the scanning session was considered necessary to: 1- familiarize them with the different types of stimulus images; 2- train them to use one word names to diagnose radiological lesions.

Initially, it was verbally explained to the participant the task s/he would face. Subsequently, lists with the name of the alterations were handed to the participant. There were three pages, each one with the list of the different types of stimuli in each category. The order of presentation of the lists was counterbalanced between participants.

A critical point in the preparation of the participants was their training to use one word names for radiological lesions. Previously, in pilot tests, we presented the one word names for lesions as a standardized list. Our initial strategy was to prime those words showing its list to the participants three times and making corrections during the training. However, we suspected that the emphasis was having an ironic effect in which the effort to focus on the 'right' responses was making competing words more available to the awareness of participants and possibly generating anxiety performance.

We changed the approach. The list of one word names for lesions was presented simply as a suggestion; it was emphasized to the participant to feel free to vocalize the word related to the alteration with which s/he was more comfortable with. Initially, in the training session, we corrected the verbalizations which were different from the standardized list; afterwards corrections during the training were made only when there was a diagnostic error.

Three different sets of 60 images with the same sequence design optimized for the experiment, including null events, were presented during the training using E-Prime 2.0. In the first sequence the image presentation was paced by the researcher. In the second sequence the participant was instructed to vocalize the responses with the mouth closed to minimize jaw and head movements. Again the researcher paced the session, changing images after the participant's response. In the third training sequence the images were presented automatically following the timing of the experiment.

Concluding the training session the participant received a written review of the instructions regarding the task and behaviour in the scanner

Radiologists involved in the pilot tests of images did not participate in the experiment proper.

**6- Debriefing protocol and calculation of the lexical semantic index**

The investigation of the lexical semantic associations during the naming of the stimuli was conducted using a detailed debriefing protocol immediately after the scanning session. For every stimulus type, in each category, the participant was asked if other words besides the one vocalized came to their awareness while naming the stimuli during the fMRI sessions. The order of questioning for stimulus category was counterbalanced to avoid order effects in recall. Participants were not informed beforehand of the debriefing protocol.

The lexical semantic association index was calculated for each category of stimulus using the following the formula:

where:

n_i_: number of types of stimulus with one or more associated words for each *i* participant.

S: number of different types of stimulus per category

T: total number of participants

**References**

1. Carreiras M, Price CJ (2008) Brain activation for consonants and vowels. Cereb Cortex 18: 1727-1735.

2. Wenzlaff RM, Wegner DM (2000) Thought suppression. Annu Rev Psychol 51: 59-91.
